# A novel *in vitro* assay model developed to measure both extracellular and intracellular acetylcholine levels for screening cholinergic agents

**DOI:** 10.1371/journal.pone.0258420

**Published:** 2021-10-12

**Authors:** Ryohei Tanaka-Kanegae, Koichiro Hamada

**Affiliations:** Saga Nutraceuticals Research Institute, Otsuka Pharmaceutical Co., Ltd., Saga, Japan; Weizmann Institute of Science, ISRAEL

## Abstract

**Background:**

Cholinergic neurons utilize choline (Ch) to synthetize acetylcholine (ACh) and contain a high-affinity Ch transporter, Ch acetyltransferase (ChAT), ACh receptors, and acetylcholinesterase (AChE). As the depletion or malfunction of each component of the cholinergic system has been reported in patients with dementia, many studies have sought to evaluate whether treatment candidates affect each of the cholinergic components. The associated changes in the cholinergic components may be reflected by intra- or extra-cellular ACh levels, with an increase in extracellular ACh levels occurring following AChE inhibition. We hypothesized that increases in intracellular ACh levels can be more sensitively detected than those in extracellular ACh levels, thereby capturing subtle effects in the cholinergic components other than AChE. The objective of this study was to test this hypothesis.

**Methods:**

We developed an *in vitro* model to measure both extracellular and intracellular ACh levels using the human cholinergic neuroblastoma cell line, LA-N-2, which have been reported to express Ch transporter, ChAT, muscarinic ACh receptor (mAChR), and AChE. With this model, we evaluated several drug compounds and food constituents reported to improve cholinergic function through various mechanisms. In addition, we conducted western blotting to identify the subtype of mAChR that is expressed on the cell line.

**Results:**

Our cell-based assay system was capable of detecting increases in extracellular ACh levels induced by an AChE inhibitor at relatively high doses, as well as increases in intracellular ACh levels following the administration of lower AChE-inhibitor doses and an mAChR agonist. Moreover, increases in intracellular ACh levels were observed even after treatment with food constituents that have different mechanisms of action, such as Ch provision and ChAT activation. In addition, we revealed that LA-N-2 cells expressed mAChR M2.

**Conclusion:**

The findings support our hypothesis and indicate that the developed assay model can broadly screen compounds from drugs to food ingredients, with varying strengths and mechanisms of action, to develop treatments for ACh-relevant phenomena, including dementia and aging-related cognitive decline.

## Introduction

Acetylcholine (ACh) is a neurotransmitter that plays crucial roles in both the central and peripheral nervous systems, and central cholinergic transmission is essential for normal cognitive processes [1]. The cholinergic system contains choline (Ch), which is the substrate for ACh synthesis; high-affinity Ch transporter, which carries Ch into cholinergic neurons; Ch acetyltransferase (ChAT), which is responsible for ACh synthesis; muscarinic and nicotinic ACh receptors (mAChRs and nAChRs, respectively); and acetylcholinesterase (AChE). The depletion or malfunction of these components has been reported in individuals experiencing cognitive decline, leading to the evaluation of whether drug candidates can affect the abundance and availability of Ch and the activity of AChE and ChAT, and elicit agonistic effects at ACh receptors [2–6]. From this perspective, numerous food constituents have been studied as therapeutic candidates, as the importance of a nutritional approach to prevent cognitive decline has become apparent [7]. Given that changes in Ch availability and activities of the individual cholinergic components may be reflected in ACh levels inside and/or outside cholinergic cells, determining whether treatment candidates increase ACh levels may represent an index when exploring cholinergic agents.

*In vivo*, brain fixation [8] and microdialysis [9] techniques are commonly used to measure ACh levels. Although ACh in the **synaptic** cleft is rapidly hydrolyzed by AChE after its transmission, brain fixation enables the quantification of intracellular and extracellular ACh, while microdialysis quantifies extracellular ACh. Considering that the main sites of ACh biosynthesis and degradation are inside and outside cholinergic cells, respectively [3], the increase in intracellular ACh levels may be detected more sensitively than that in extracellular ACh levels. We, therefore, consider that an *in vitro* model capable of measuring intracellular ACh separately from extracellular ACh could serve as a promising tool to broadly screen cholinergic agents, especially food components with mild cholinergic activities. However, no such model exists, although a substantial number of *in vitro* models have been developed focusing on single cholinergic components such as AChE, ChAT, and AChRs [5, 10–13].

We further hypothesized that the detection of increases in extracellular ACh levels could be applied to determine the activity of AChE-inhibiting drugs, whereas the detection of increases in intracellular ACh levels would be sensitive enough to determine the subtle effects elicited by food constituents via various mechanisms other than AChE inhibition. To test this hypothesis, we developed an *in vitro* model and assessed several drug compounds and food components with a known capacity to enhance cholinergic function through different mechanisms, including AChE inhibition (physostigmine [14], delphinidin [15], and black ginger extract [16]), Ch provision (glycerophosphocholine [17] and lysophosphatidylcholine (LPC) [18]), ChAT activation (luteolin [19] and nobiletin [20]), and AChR activation (muscarine and cytisine [21]).

## Materials and methods

### Reagents

Dulbecco’s Modified Eagle’s Medium (DMEM)/Nutrient Mixture F-12 Ham (Ham’s F-12) (D8062), physostigmine, and (+)-muscarine chloride were purchased from Sigma-Aldrich (St. Louis, MO, USA). Cytisine was obtained from LKT Laboratories, Inc. (St. Paul, MN, USA). DMEM/Ham’s F-12 without Ch chloride was obtained from Cell Science & Technology Inst., Inc. (Miyagi, Japan). Fetal bovine serum (FBS) was purchased from Nichirei Biosciences Inc. (Tokyo, Japan). Penicillin and streptomycin were purchased from Thermo Fisher Scientific, Inc. (Waltham, MA, USA). ACh bromide, Ch chloride, LPC from egg yolk, and luteolin were purchased from Wako Pure Chemical Industries Ltd. (Osaka, Japan). Isopropylhomocholine (IPHC) was provided by Eicom Corporation (Kyoto, Japan). Delphinidin chloride was purchased from Tokiwa Phytochemical Co., Ltd. (Chiba, Japan), and black ginger extract was obtained from Maruzen Pharmaceuticals Co., Ltd. (Hiroshima, Japan). Nobiletin was purchased from Indofine Chemical Company, Inc. (Hillsborough, NJ, USA). Other food constituents evaluated in the present study are listed in S1 Table. Lysis buffer was obtained from Bio-Rad Laboratories, Inc. (Hercules, CA, USA). Rabbit anti-mAChR M2 antibody (Ab) (ab109226) was purchased from Abcam (Cambridge, UK) and anti-rabbit Ab horseradish peroxidase (HRP)-linked IgG Ab was obtained from Cell Signaling Technology (Beverly, MA, USA). Amersham enhanced chemiluminescence (ECL) blocking agent and ECL western blotting detection reagents were purchased from Cytiva (Marlborough, MA, USA). Mouse brain tissue lysate was obtained from Abcam. All reagents and chemicals used were of reagent grade.

### Cell culture

We used the human neuroblastoma cell line LA-N-2 for the *in vitro* model as it has a cholinergic phenotype and expresses Ch transporter, ChAT, mAChR, and AChE [18, 22–27], which makes it suitable for evaluating the effects of the candidates on intracellular and extracellular ACh levels. The cell line was purchased from the **European Collection of Authenticated Cell Cultures**. DMEM/Ham’s F-12 supplemented with 10% heat-inactivated FBS, penicillin (100 U/mL), and streptomycin (100 μg/mL) was used for cell culture, whereas DMEM/Ham’s F-12 without Ch chloride supplemented with 2% heat-inactivated FBS was used as an assay medium. The cells were incubated at 37°C in a humidified atmosphere of 95% air and 5% CO_2_. The number of passages of the cells used for assays ranged from 15 to 25.

### Assays

LA-N-2 cells were seeded at 4.0 × 10^5^ cells/well in a 24-well plate and cultured until sub-confluent. During the assay, the culture medium was replaced with 1 mL of the aforementioned assay medium with the test compound dissolved in water, ethanol, or dimethyl sulfoxide (DMSO) (final concentration: 0.1% (*v/v*) solvent). Test compounds were used in the concentration range at which they did not show any cytotoxicity. After 5 h of incubation at 37°C, the medium was collected as the extracellular fraction, and the cells were rinsed with phosphate-buffered saline (PBS) and scraped with 200 μL of 0.1 M ice-cold perchloric acid to deactivate proteins that could affect Ch and ACh levels. The residual cells and wells were washed with 200 μL of perchloric acid; the rinse solution was mixed with the aforementioned lysate in perchloric acid (the total volume is 400 μL) and stored as the intracellular fraction. The collected culture medium and cell lysates were stored at -30°C until Ch and ACh measurement.

### Choline and acetylcholine measurement

We decided to employ electrochemical detection combined with high-performance liquid chromatography (HPLC) to measure Ch and ACh levels owing to its practical advantages, including high sensitivity and selectivity, rapid response, and operational simplicity [28]. We then followed a previously described quantification method [29] with minor modifications. The HPLC system consisted of a pump, column oven, electrochemical detector (ECD) (HTEC-500; Eicom Corporation), data processor (D-7000; Hitachi Ltd., Tokyo, Japan), autosampler (L-7200; Hitachi Ltd.), guard column (CH-GEL, 3.0 mm internal diameter (ID); Eicom Corporation), separation column (Eicompak AC-GEL, 2.0 mm ID × 150 mm; Eicom Corporation), and enzyme column immobilized with AChE and Ch oxidase (AC-ENZYM II, 1.0 mm ID × 4.0 mm; Eicom Corporation). The temperature of the column oven was maintained at 33°C, and a platinum working electrode was used at 450 mV versus a silver (Ag)/silver chloride (AgCl) reference electrode (both electrodes from Eicom Corporation). A mobile phase containing 50 mM potassium bicarbonate, 2.5 mmol/L (M) sodium 1-decanesulfonate, and 134 μM EDTA·2Na was used under isocratic conditions at a flow rate of 150 μL/min.

**Fig 1:**
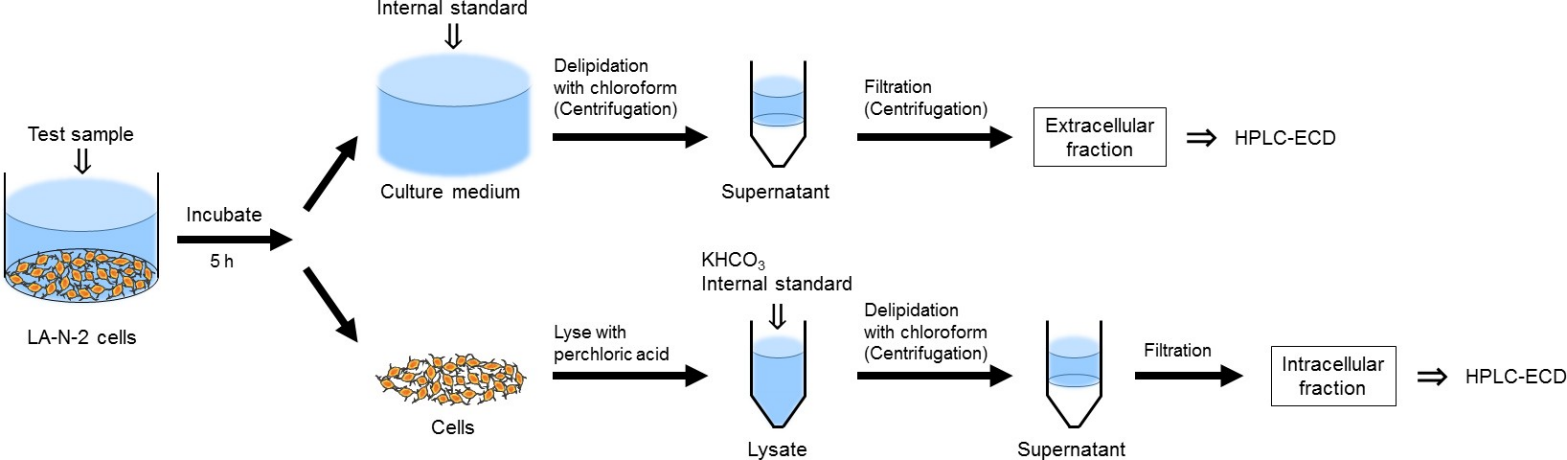
A schematic diagram of the new *in vitro* assay system. The procedures to measure extracellular and intracellular acetylcholine levels are illustrated.

The collected samples in assays were pretreated for the HPLC analysis. The cell lysates in perchloric acid were sonicated for 3 min (Bioruptor; Tosyo Denki, Kanagawa, Japan) and centrifuged (15,000 × *g*, 15 min, 4°C). The supernatants were collected and neutralized by adding 1 M potassium bicarbonate. After adding 45 pmol IPHC as an internal standard, the supernatants were mixed with chloroform and vigorously agitated. Delipidation was completed by centrifugation (15,000 × *g*, 10 min, 4°C) and filtration of the supernatants through a 0.2-μm polytetrafluoroethylene (PTFE) membrane (Millex-LG; Millipore Corporation, Billerica, MA, USA). The culture media were mixed with IPHC and subjected to the same delipidation process as the cell lysates. Filtration was carried out using a 5-kDa cut-off filter (**Ultrafree**^®^ **MC-PLHCC;** Human Metabolome Technologies, Inc., Yamagata, Japan), and the filtrates were injected into the HPLC system. Stock solutions containing Ch and ACh were prepared using 20 mM phosphate buffer containing 20 mM EDTA·2Na, and then aliquoted and preserved at -80°C. For each assay, the stock solution was serially diluted, and IPHC was added to each diluted solution. The ratios of Ch to IPHC and ACh to IPHC were used to construct standard curves; the quantitative range for Ch and ACh was 20 nM to 2 μM. The Ch and ACh content in the samples was recalculated as a percentage of the Ch or ACh level in the test group to that in the vehicle-treated (control) group for interexperimental comparison. For treatments that increased ACh levels, we repeated the assay at least once to confirm reproducibility. A schematic diagram of the new assay system is shown in Fig 1.

### Western blotting

LA-N-2 cells (4.0 × 10^6^ cells) were seeded into 6-mm dishes and cultured until sub-confluent. Then, cells were treated with or without the assay medium for 5 h. Next, they were washed with PBS twice and lysed in a lysis buffer including 2 mM phenylmethylsulfonyl fluoride. The lysate was sonicated and centrifuged (4,500 × *g*, 20 min, 4°C), and the supernatant was collected. Denatured proteins were separated by sodium dodecyl sulfate-polyacrylamide gel electrophoresis (SDS-PAGE) using 10% polyacrylamide gels, and then transferred to polyvinylidene fluoride (PVDF) membranes (Bio-Rad). After blocking with 2% Amersham ECL blocking agent in Tris-buffered saline with **Tween**-20, the membrane was treated with rabbit anti-mAChR M2 Ab (1:1000) over night at 4°C, followed by the corresponding HRP-conjugated secondary Ab (1:1000) for 2 h at room temperature. The blots were developed using ECL western blotting detection reagents. Mouse brain tissue lysate was used as a positive control.

### Statistical analysis

For each assay, significant differences between the control and test groups were evaluated using the unpaired *t*-test (two-tailed). For multiple comparisons, Dunnett’s *post hoc* test was performed after one-way analysis of variance (ANOVA). Differences with probability (*p*) values < 0.05 were considered statistically significant. SAS 9.4 (SAS Institute Inc., Cary, NC, USA) was used for statistical analyses.

## Results

In LA-N-2 cells treated with physostigmine, the intracellular ACh levels increased dose-dependently, with significant differences from those of the control at doses higher than 500 nM (107.8%, *p* = 0.024 for 500 nM; 109.2%, *p* = 0.009 for 5 μM; and 113.4%, *p* < 0.001 for 50 μM), whereas the intracellular Ch levels did not change (Table 1A, Assay 1). Physostigmine at doses higher than 5 μM also led to the detection of ACh in the culture media (extracellular fraction), but not in the control group. In the control group, the extracellular Ch levels were higher than the intracellular Ch levels 5 h after replacing the culture medium with the assay medium (Table 1B, Assay 1). Subsequently, we tested delphinidin and black ginger extract. We observed that 100 μM delphinidin and 100 μg/mL black ginger extract reproducibly increased intracellular ACh levels up to 110.9% and 111.1% compared with the control, respectively. In contrast, the intracellular Ch levels did not significantly change after these treatments (Table 1A, Assays 2 and 3). Further, delphinidin and black ginger extract in the tested concentration ranges did not lead to ACh detection or any change in Ch levels in the extracellular fraction (Table 1B, Assays 2 and 3).

**Table 1:** Changes in the choline and acetylcholine levels in LA-N-2 cells after treatment with physostigmine, delphinidin, and black ginger extract, which have been reported to have AChE-inhibiting activity.

(A) Intracellular							
		Ch			ACh		
		(μM)	(pmol)	(%)	(μM)	(pmol)	(%)
Assay 1	Control	0.181 ± 0.073	72.3 ± 29.4	100.0 ± 40.6	0.331 ± 0.018	132.2 ± 7.3	100.0 ± 5.6
	5 nM physostigmine	0.164 ± 0.060	65.6 ± 24.1	90.7 ± 33.3	0.329 ± 0.008	131.7 ± 3.2	99.6 ± 2.4
	50 nM physostigmine	0.191 ± 0.062	76.6 ± 24.6	105.8 ± 34.0	0.353 ± 0.007	141.1 ± 2.6	106.7 ± 2.0
	500 nM physostigmine	0.173 ± 0.077	69.3 ± 30.8	95.8 ± 42.6	0.356 ± 0.003	142.6 ± 1.2	107.8 ± 0.9 *
	5 μM physostigmine	0.203 ± 0.062	81.2 ± 24.7	112.2 ± 34.1	0.361 ± 0.008	144.3 ± 3.3	109.2 ± 2.5 **
	50 μM physostigmine	0.175 ± 0.050	70.1 ± 20.2	96.9 ± 27.9	0.375 ± 0.004	150.0 ± 1.6	113.4 ± 1.2 **
Assay 2	Control	0.143 ± 0.039	57.4 ± 15.7	100.0 ± 27.3	0.389 ± 0.009	155.7 ± 3.5	100.0 ± 2.2
	25 μM delphinidin	0.165 ± 0.070	66.0 ± 28.0	115.0 ± 48.8	0.415 ± 0.011	165.9 ± 4.6	106.6 ± 2.9
	50 μM delphinidin	0.168 ± 0.055	67.4 ± 22.1	117.4 ± 38.5	0.415 ± 0.007	165.9 ± 2.6	106.6 ± 1.7
	100 μM delphinidin	0.178 ± 0.085	71.1 ± 33.9	124.0 ± 59.1	0.432 ± 0.016	172.6 ± 6.2	110.9 ± 4.0 **
Assay 3	Control	0.198 ± 0.092	79.1 ± 36.9	100.0 ± 46.6	0.452 ± 0.022	180.9 ± 8.9	100.0 ± 4.9
	25 μg/mL black ginger	0.205 ± 0.060	81.9 ± 24.1	103.5 ± 30.5	0.454 ± 0.023	181.5 ± 9.1	100.3 ± 5.1
	50 μg/mL black ginger	0.178 ± 0.068	71.0 ± 27.1	89.8 ± 34.3	0.458 ± 0.023	183.3 ± 9.2	101.4 ± 5.1
	100 μg/mL black ginger	0.177 ± 0.018	70.6 ± 7.2	89.4 ± 9.1	0.502 ± 0.013	201.0 ± 5.3	111.1 ± 2.9 *
(B) Extracellular							
		Ch			ACh		
		(μM)	(pmol)	(%)	(μM)	(pmol)	(%)
Assay 1	Control	3.16 ± 0.12	3158 ± 123	100.0 ± 3.9	ND	ND	-
	5 nM physostigmine	3.20 ± 0.08	3204 ± 76	101.4 ± 2.4	ND	ND	-
	50 nM physostigmine	3.24 ± 0.04	3239 ± 45	102.5 ± 1.4	ND	ND	-
	500 nM physostigmine	3.35 ± 0.19	3350 ± 194	106.1 ± 6.1	ND	ND	-
	5 μM physostigmine	3.33 ± 0.15	3326 ± 155	105.3 ± 4.9	0.0310 ± 0.0012	31.0 ± 1.2	-
	50 μM physostigmine	2.86 ± 0.13	2859 ± 126	90.5 ± 4.0	0.0339 ± 0.0026	33.9 ± 2.6	-
Assay 2	Control	3.29 ± 0.11	3289 ± 109	100.0 ± 3.3	ND	ND	-
	25 μM delphinidin	3.23 ± 0.18	3226 ± 181	98.1 ± 5.5	ND	ND	-
	50 μM delphinidin	3.42 ± 0.08	3418 ± 75	103.9 ± 2.3	ND	ND	-
	100 μM delphinidin	3.47 ± 0.31	3469 ± 306	105.5 ± 9.3	ND	ND	-
Assay 3	Control	3.81 ± 0.36	3811 ± 356	100.0 ± 9.3	ND	ND	-
	25 μg/mL black ginger	3.92 ± 0.56	3919 ± 565	102.8 ± 14.8	ND	ND	-
	50 μg/mL black ginger	3.89 ± 0.39	3891 ± 391	102.1 ± 10.3	ND	ND	-
	100 μg/mL black ginger	4.17 ± 0.44	4166 ± 442	109.3 ± 11.6	ND	ND	-

The values are expressed as absolute (μM and pmol) and relative to vehicle control (%). Data are presented as mean ± standard deviation (SD), n = 3 in all groups

*p < 0.05

**p < 0.01. ACh, acetylcholine; AChE, acetylcholinesterase; Ch, choline; ND, not detected.

Next, we checked the response of LA-N-2 cells to Ch and Ch-containing compounds. After the incubation of LA-N-2 cells with 100 μM Ch, the intracellular Ch and ACh levels increased to 984.7% and 179.5% compared with the control, respectively (Table 2, Assay 1). The extracellular Ch level was above the quantitative limit, and ACh was not detected (S1 Dataset). When the cells were treated with LPC at concentrations between 3.125 and 25 μg/mL, both the intracellular Ch and ACh levels increased in a concentration-dependent manner. Ch levels reached statistical significance when treated with LPC at concentrations higher than 12.5 μg/mL (860.1%, *p* = 0.014 for 12.5 μg/mL; and 1259.7%, *p* < 0.001 for 25 μg/mL), and ACh levels at concentrations higher than 6.25 μg/mL (122.0%, *p* = 0.009 for 6.25 μg/mL; 129.8%, *p* < 0.001 for 12.5 μg/mL; and 147.6%, *p* < 0.001 for 25 μg/mL; Table 2, Assay 2). However, ACh was not detected in the extracellular fraction after LPC treatment. In addition, glycerophosphocholine and phosphatidylcholine did not increase the intracellular Ch or ACh levels; ACh was not detected in the extracellular fraction after the treatments (S1 Dataset).

**Table 2:** Changes in the choline and acetylcholine levels in LA-N-2 cells after treatment with choline and lysophosphatidylcholine that have a Ch moiety.

Intracellular							
		Ch			ACh		
		(μM)	(pmol)	(%)	(μM)	(pmol)	(%)
Assay 1	Control	0.079 ± 0.017	31.6 ± 6.9	100.0 ± 22.0	0.281 ± 0.024	112.4 ± 9.6	100.0 ± 8.5
	100 μM choline	0.778 ± 0.154	311.2 ± 61.7	984.7 ± 195.3 **	0.504 ± 0.012	201.7 ± 4.7	179.5 ± 4.1 **
Assay 2	Control	0.116 ± 0.028	46.4 ± 11.2	100.0 ± 24.2	0.231 ± 0.013	92.4 ± 5.4	100.0 ± 5.8
	3.125 μg/mL LPC	0.249 ± 0.102	99.5 ± 40.8	214.7 ± 87.9	0.265 ± 0.008	105.8 ± 3.2	114.6 ± 3.5
	6.25 μg/mL LPC	0.443 ± 0.236	177.0 ± 94.5	381.9 ± 203.8	0.282 ± 0.015	112.7 ± 5.8	122.0 ± 6.3 **
	12.5 μg/mL LPC	0.997 ± 0.366	398.7 ± 146.5	860.1 ± 316.1 *	0.300 ± 0.015	119.9 ± 5.8	129.8 ± 6.3 **
	25 μg/mL LPC	1.460 ± 0.694	583.9 ± 277.6	1259.7 ± 598.9 **	0.341 ± 0.036	136.3 ± 14.6	147.6 ± 15.8 **

The values are expressed as absolute (μM and pmol) and relative to vehicle control (%). Data are presented as mean ± standard deviation (SD), n = 3 in all groups

*p < 0.05

**p < 0.01. ACh, acetylcholine; Ch, choline; LPC, lysophosphatidylcholine.

Subsequently, we assessed luteolin and nobiletin using the cell-based assay system. Although extracellular ACh was not detected, intracellular ACh levels significantly increased after treatment with 50 and 100 μM luteolin (111.6%, *p* = 0.006 for 50 μM; and 128.3%, *p* < 0.001 for 100 μM), and 100 μM nobiletin (108.6%, *p* = 0.025). There were no significant changes in intracellular Ch levels (Table 3).

**Table 3:** Changes in the choline and acetylcholine levels in LA-N-2 cells after treatment with luteolin and nobiletin, which have been reported to have ChAT-increasing activity.

Intracellular							
		Ch			ACh		
		(μM)	(pmol)	(%)	(μM)	(pmol)	(%)
Assay 1	Control	0.202 ± 0.073	80.9 ± 29.2	100.0 ± 36.1	0.450 ± 0.003	180.1 ± 1.2	100.0 ± 0.7
	25 μM luteolin	0.207 ± 0.084	82.6 ± 33.5	102.1 ± 41.5	0.448 ± 0.001	179.0 ± 0.5	99.4 ± 0.3
	50 μM luteolin	0.211 ± 0.102	84.5 ± 40.8	104.4 ± 50.4	0.502 ± 0.027	201.0 ± 10.9	111.6 ± 6.0
	100 μM luteolin	0.223 ± 0.021	89.1 ± 8.4	110.2 ± 10.3	0.578 ± 0.009	231.1 ± 3.6	128.3 ± 2.0 **
Assay 2	Control	0.143 ± 0.053	57.1 ± 21.3	100.0 ± 37.3	0.515 ± 0.014	206.1 ± 5.7	100.0 ± 2.8
	100 μM nobiletin	0.113 ± 0.014	45.1 ± 5.5	78.9 ± 9.7	0.560 ± 0.017	223.9 ± 6.7	108.6 ± 3.3 *

The values are expressed as absolute (μM and pmol) and relative to vehicle control (%). Data are presented as mean ± standard deviation (SD), n = 3 in all groups

*p < 0.05

**p < 0.01. ACh, acetylcholine; Ch, choline; ChAT, choline acetyltransferase.

**Fig 2:**
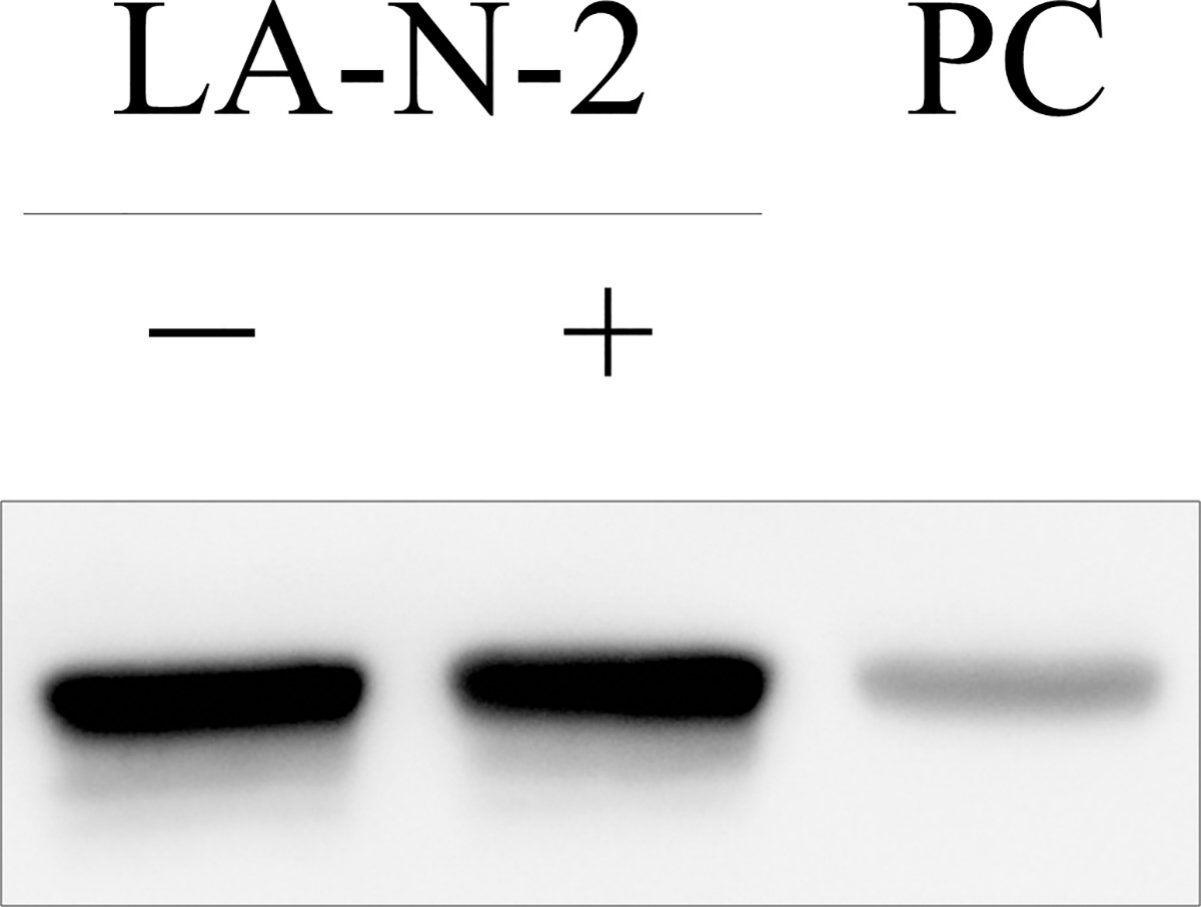
The expression of muscarinic acetylcholine receptor M2 on LA-N-2 cells. LA-N-2 cells were lysed before (-) or after (+) the treatment with the assay medium for 5 h and subjected to SDS-PAGE along with mouse brain tissue lysate as a positive control (PC). Lysates/proteins at 10 μg per lane.

Finally, we treated LA-N-2 cells with 100 μM muscarine, an mAChR agonist. Although extracellular ACh was not detected, intracellular ACh levels significantly increased up to 122.5% compared with the control (*p* = 0.038); intracellular Ch levels did not change after muscarine treatment (Table 4). Other experiments showed that treatment with up to 5 μM cytisine, an nAChR agonist, neither changed ACh nor Ch levels (S1 Dataset). Moreover, western blotting revealed that LA-N-2 cells expressed mAChR M2, even after treatment with the assay medium for 5 h (Fig 2).

**Table 4:** Changes in the choline and acetylcholine levels in LA-N-2 cells after treatment with muscarine, a muscarinic acetylcholine receptor agonist.

Intracellular						
	Ch			ACh		
	(μM)	(pmol)	(%)	(μM)	(pmol)	(%)
Control	0.0652 ± 0.0196	26.1 ± 7.8	100.0 ± 30.0	0.184 ± 0.023	73.6 ± 9.4	100.0 ± 12.8
100 μM muscarine	0.0617 ± 0.0153	24.7 ± 6.1	94.6 ± 23.5	0.225 ± 0.001	90.1 ± 0.2	122.5 ± 0.3 *

The values are expressed as absolute (μM and pmol) and relative to vehicle control (%). Data are presented as mean ± standard deviation (SD), n = 3 in all groups

*p < 0.05. ACh, acetylcholine; Ch, choline.

We have listed drug compounds and food constituents that increased ACh levels in our cell-based assay in Table 5 with their reported mechanisms of action on the cholinergic system, and the other drug compounds and food constituents, which did not, in Table 6.

**Table 5:** Drug compounds and food constituents that increased extracellular and/or intracellular ACh levels in our cell-based assay.

Reported mechanism of action on the cholinergic system	Name	Drug/food	Concentration used in assay	Fraction where the increase was detected
AChE inhibition	Physostigmine	Drug	5 nM–50 μM	Extracellular and intracellular
	Delphinidin chloride	Food	25–100 μM	Intracellular
	Black ginger extract	Food	25–100 μg/mL	Intracellular
Ch (ACh precursor) provision	Ch chloride	Food	100 μM	Intracellular
	LPC	Food	3.125–25 μg/mL	Intracellular
ChAT activation	Luteolin	Food	25–100 μM	Intracellular
	Nobiletin	Food	100 μM	Intracellular
mAChR activation	(+)-Muscarine chloride	Drug	100 μM	Intracellular

Abbreviations: ACh, acetylcholine; AChE, acetylcholinesterase; Ch, choline; ChAT, choline acetyltransferase; LPC, lysophosphatidylcholine; mAChR, muscarinic acetylcholine receptor.

**Table 6:** A drug compound and food constituents that did not increase extracellular or intracellular ACh levels in our cell-based assay.

Name	Drug/food	Concentration used in assay
Cytisine	Drug	50 nM–5 μM
Glycerophosphocholine	Food	100 μg/mL
Arachidonic acid	Food	20 μg/mL
Astaxanthin	Food	100 μM
L-Carnitine	Food	100 μM
Citrulline	Food	100 μM
Curcumin	Food	20 μM
Cyanidin chloride	Food	100 μM
Cyanocobalamin	Food	100 μM
Docosahexaenoic acid	Food	20 μg/mL
Eicosapentaenoic acid	Food	20 μg/mL
Ferulic acid	Food	100 μM
Glycine	Food	100 μM
Lutein	Food	100 μM
Methylcobalamin	Food	100 μM
Octanoic acid	Food	100 μg/mL
Phosphatidylcholine from egg yolk	Food	100 μg/mL
Phosphatidylethanolamine, dimyristoyl	Food	100 μg/mL
Phosphatidylethanolamine, dioleoyl	Food	100 μg/mL
Phosphatidylethanolamine, dipalmitoyl	Food	100 μg/mL
Phosphatidylethanolamine, distearoyl	Food	100 μg/mL
Phosphatidylserine	Food	100 μg/mL
Pyrroloquinoline quinone	Food	100 μM
L-Serine	Food	100 μM
cis-15-Tetracosenoic acid	Food	100 μg/mL
Zeaxanthin	Food	100 μM

## Discussion

When LA-N-2 cells were incubated in the assay medium for 5 h as the control, the concentration of extracellular Ch was quantified to be 3–4 μM (Table 1B). Tucek reported that the concentration of extracellular Ch in the human brain is similar to that in the cerebrospinal fluid, within the range of 0.4–5.1 μM, and this notion has well been supported by microdialysis studies [30, 31]. Importantly, the concentration of extracellular Ch observed in our study fell within this range, which indicates the ability of this system to simulate the cholinergic metabolic system in physiological conditions.

ACh was not detected in the extracellular fraction in the control group (Table 1B). This supports the finding that this cell type expresses AChE [24]. Although the cholinergic phenotype of LA-N-2 cells is known to be enhanced when differentiated [24, 26], the cell line without differentiation seemed to produce a sufficient amount of ChAT for intracellular ACh to be produced and AChE for extracellular ACh to be degraded. Therefore, we prioritized a quick screen system set up and decided to perform assays without differentiation.

When LA-N-2 cells were treated with physostigmine, a commonly used AChE inhibitor, at doses higher than 5 μM, extracellular ACh was successfully detected. Given its short half-life [14], it could be possible to use an even lower concentration of physostigmine to detect extracellular ACh if the incubation time is optimized. However, 5 μM physostigmine is commonly used when performing microdialysis to increase the basal ACh level up to a detectable magnitude [32]. In addition, Lau et al. reported that their *in vitro* model using lung cancer cells could detect the increase in extracellular ACh levels after neostigmine treatment. Given that they successfully detected this increase using 50 μM neostigmine but not 20 μM [33], and that the half-maximal inhibitory concentration of neostigmine toward AChE is similar to that of physostigmine [32], we consider that our model can sensitively detect the extracellular ACh following AChE inhibition. Interestingly, using physostigmine, the detection of extracellular ACh coincided with the increase in intracellular ACh levels; this increase was observed at a ten times lower dose of physostigmine (Table 1, Assay 1).

Delphinidin, an anthocyanidin commonly present in pigmented fruits and vegetables [15], and black ginger (*Kaempferia parviflora*) extract have also been reported to have an inhibitory effect on AChE [15, 16]. Treatments with these two food components increased intracellular ACh levels, although ACh was not detected in the extracellular fraction (Table 1, Assays 2 and 3). These findings support the possibility that the increase in intracellular ACh levels is more easily detected than that in extracellular ACh levels. Moreover, the findings suggest that our cell-based assay model is sensitive enough to detect the increase in intracellular ACh levels induced by food components. Given that delphinidin and black ginger extract improve cognitive function *in vivo* [15, 34], an increase in the intracellular ACh levels may serve as an indicator of cognitive improvement. For these reasons, we decided to proceed with the assays with a focus on intracellular ACh.

When Ch was added to the assay medium, it was taken up and converted to ACh in the cells (Table 2, Assay 1); this supports the existence of Ch transporter and ChAT in LA-N-2 cells. LPC, which constitutes enzyme-modified lecithin and is used as a food emulsifier owing to its amphipathic nature [35], has been shown to be taken up by and converted to ACh via Ch in LA-N-2 cells [18]. This was confirmed with 3.125 to 25 μg/mL LPC in our experimental model, and 25 μg/mL LPC evoked higher intracellular Ch levels than 100 μM Ch (Table 2, Assays 1 and 2). This finding can be explained by the intermediate-affinity uptake of Ch by LA-N-2 cells [27] and the detergent-like property of LPC; 50 μg/mL LPC caused cytotoxicity and decreased intracellular ACh levels (S1 Dataset).

Glycerophosphocholine and phosphatidylcholine caused no significant changes in intracellular Ch or ACh levels (Table 6, S1 Dataset). As LA-N-2 cells have been reported to have glycerophosphodiester phosphodiesterase activity [24], which hydrolyzes glycerophosphocholine to Ch, it seems that glycerophosphocholine was not incorporated into the cells nor degraded by the enzyme. Moreover, LA-N-2 cells have been shown to express phospholipase D to utilize phosphatidylcholine in the cell membrane [18]. However, as the cell membrane was impermeable to the phosphatidylcholine in the medium, its utilization as an ACh precursor was prevented. Similarly, we assume that phosphatidylserine, which has been shown to reverse ACh levels in aged animals [36], failed to increase ACh levels in our cell-based assay (Table 6, S1 Dataset) owing to the lack of related metabolic enzymes and its inability to cross the cell membrane from the medium.

The intracellular ACh levels increased after treatment with luteolin and nobiletin (Table 3). Luteolin is a flavonoid that is present in fruits and vegetables such as celery, chrysanthemum flower, sweet bell pepper, carrot, onion leaf, broccoli, and parsley [37]; the oral administration of luteolin has been reported to increase ACh levels in the brain of amyloid β-infused rats through ChAT activation [19]. Nobiletin, a citrus flavonoid known to improve cognitive function *in vivo* [38], has also been reported to increase ACh levels via the upregulation of ChAT expression [20]. These findings suggest that the increase in intracellular ACh levels observed after treatments with these flavonoids resulted from the upregulation of ChAT.

The cell line showed a significant increase in intracellular ACh levels when incubated with muscarine (Table 4). To the best of our knowledge, previous studies have suggested the existence of mAChR on LA-N-2 cells but have not identified the subtype expressed [25]. Therefore, we performed western blotting and showed the constant expression of mAChR M2 on the cells (Fig 2). The increase in intracellular ACh levels following muscarine stimulation may be partly mediated by mAChR M2 because the activation of this subtype leads to the feedback inhibition of ACh release and accumulation of ACh in neurons [39]. Importantly, our result demonstrated that an increase in the intracellular ACh levels owing to stimulation by mAChR agonists could be detected by our assay system. In contrast, another ACh receptor type, nAChR, may not be expressed on LA-N-2 cells, which was supported by our data; cytisine, a nicotinic agonist [21], did not alter ACh or Ch levels (Table 6, S1 Dataset).

As shown above, our cell-based assay system could detect an increase in extracellular ACh levels by an AChE-inhibiting drug at relatively high doses. In addition, the developed model could detect an increase in intracellular ACh levels by lower doses of the AChE-inhibiting drug and an mAChR agonist, and the increase was observed even when treated with food constituents that have different mechanisms of action, such as Ch provision and ChAT activation. We consider that compared with previously reported *in vitro* models that evaluate a single component of the cholinergic system, the developed model makes it possible to more broadly screen lead compounds that have different strengths of activity and mechanisms of action by evaluating both extracellular and intracellular ACh. Although the development of cholinergic drugs has been highly focused on ACh inhibition and many AChE inhibitors have been developed [10, 11, 40], food constituents that are expected to counteract cognitive decline have various mechanisms besides AChE inhibition, as shown above [15, 16, 19, 20, 34, 38]. Therefore, the developed assay model may be particularly effective to screen food constituents with mild cholinergic activities.

According to the amyloid hypothesis, the accumulation of amyloid β in the brain is the primary factor driving the pathogenesis of Alzheimer’s disease. Although a treatment approach based on this hypothesis is one of the most convincing approaches [41], multidirectional approaches may be needed for the management of dementia given the complex pathogenesis [42]. In addition, the interplay between amyloid β and the cholinergic system has been studied; the results showed that the activation of mAChR can shift the processing of amyloid β precursor protein toward the nonamyloidogenic pathway [3]. From these perspectives, the practical applicability of the developed assay model for screening cholinergic agents is evident.

Our study had the following limitations. First, we did not measure the activity of components of the cholinergic system (e.g., AChE) or investigate the interactions between the test molecules and cholinergic markers. Therefore, we cannot exclude the possibility that a test compound may have different mechanisms of action (e.g., inhibiting AChE and activating ChAT) simultaneously. Second, we used doses of drugs and food constituents up to 100 μM or 100 μg/mL because our main objective was to compare the response of intracellular and extracellular ACh. Given the metabolic process and existence of the blood–brain barrier, it is unlikely for some drugs and food constituents evaluated in this study to reach the brain at such doses [43]. The assay model described here should serve as a preliminary *in vitro* screen, and further *in vivo* and clinical studies are needed to clarify whether the candidates selected using this screening model could improve ACh transmission, neural plasticity, and even cognitive function.

In conclusion, this is the first study to report an assay model using LA-N-2 cells where both extracellular and intracellular ACh can be quantified. The simple and quick method enabled us to screen cholinergic agents, and the values were comparable and reproducible between assays when expressed as levels relative to those of the control. Notably, the increase in ACh levels by food constituents with different mechanisms of action on the cholinergic system was sensitively detected in the intracellular fraction. We propose that the developed screening system can be used in the first step of the development of new functional foods, as well as drugs, to treat ACh-relevant phenomena such as dementia and aging-related cognitive decline.

## Supporting information

S1 Table List of drug compounds and food constituents evaluated in our cell-based assay. (DOCX)

S1 Dataset A dataset for choline and acetylcholine quantification. (XLSX)

S1 Raw image The raw image of western blot. Fig 1 was generated from this raw image. We evaluated different lysates of LA-N-2 cells that were harvested another day, and confirmed the reproducibility of the expression of mAChR M2.
(TIF)

S2 Raw image The raw image of western blot. The same membrane as S1 Raw image but with longer exposure time to increase the intensity of bands of the molecular weight marker.
(TIF)

## References

[1] Ballinger EC, Ananth M, Talmage DA, Role LW. Basal forebrain cholinergic circuits and signaling in cognition and cognitive decline. Neuron. 2016;91(6):1199–1218. doi: 10.1016/j.neuron.2016.09.006 27657448 PMC5036520

[2] Amenta F, Tayebati SK. Pathways of acetylcholine synthesis, transport and release as targets for treatment of adult-onset cognitive dysfunction. Curr Med Chem. 2008;15(5):488–498. doi: 10.2174/092986708783503203 18289004

[3] Ferreira-Vieira TH, Guimaraes IM, Silva FR, Ribeiro FM. Alzheimer’s disease: targeting the cholinergic system. Curr Neuropharmacol. 2016;14(1):101–115. doi: 10.2174/1570159x13666150716165726 26813123 PMC4787279

[4] Gareri P, Castagna A, Cotroneo AM, Putignano S, De Sarro G, Bruni AC. The role of citicoline in cognitive impairment: pharmacological characteristics, possible advantages, and doubts for an old drug with new perspectives. Clin Interv Aging. 2015;10:1421–1429. doi: 10.2147/CIA.S87886 26366063 PMC4562749

[5] Contestabile A, Ciani E, Contestabile A. The place of choline acetyltransferase activity measurement in the “cholinergic hypothesis” of neurodegenerative diseases. Neurochem Res. 2008;33(2):318–327. doi: 10.1007/s11064-007-9497-4 17940885

[6] Scarpa M, Hesse S, Bradley SJ. M1 muscarinic acetylcholine receptors: A therapeutic strategy for symptomatic and disease-modifying effects in Alzheimer’s disease? Adv Pharmacol. 2020;88:277–310. doi: 10.1016/bs.apha.2019.12.003 32416870

[7] Muñoz Fernández SS, Lima Ribeiro SM. Nutrition and Alzheimer disease. Clin Geriatr Med. 2018;34(4):677–697. doi: 10.1016/j.cger.2018.06.012 30336995

[8] Bertrand N, Beley P, Beley A. Brain fixation for acetylcholine measurements. J Neurosci Methods. 1994;53(1):81–85. doi: 10.1016/0165-0270(94)90147-3 7990517

[9] Pepeu G, Giovannini MG. Changes in acetylcholine extracellular levels during cognitive processes. Learn Mem. 2004;11(1):21–27. doi: 10.1101/lm.68104 14747513

[10] Li S, Huang R, Solomon S, Liu Y, Zhao B, Santillo MF, et al. Identification of acetylcholinesterase inhibitors using homogenous cell-based assays in quantitative high-throughput screening platforms. Biotechnol J. 2017;12(5):1600715. doi: 10.1002/biot.201600715 28294544

[11] Santillo MF, Liu Y. A fluorescence assay for measuring acetylcholinesterase activity in rat blood and a human neuroblastoma cell line (SH-SY5Y). J Pharmacol Toxicol Methods. 2015;76:15–22. doi: 10.1016/j.vascn.2015.07.002 26165232

[12] Pulido-Rios MT, Steinfeld T, Armstrong S, Watson N, Choppin A, Eglen R, et al. In vitro isolated tissue functional muscarinic receptor assays. Curr Protoc Pharmacol. 2010;Chapter 4:Unit 4.15. doi: 10.1002/0471141755.ph0415s48 22294371

[13] Dunlop J, Roncarati R, Jow B, Bothmann H, Lock T, Kowal D, et al. In vitro screening strategies for nicotinic receptor ligands. Biochem Pharmacol. 2007;74(8):1172–1181. doi: 10.1016/j.bcp.2007.07.006 17706607

[14] Coelho F, Birks J. Physostigmine for Alzheimer’s disease. Cochrane Database Syst Rev. 2001;2001(2):CD001499. doi: 10.1002/14651858.CD001499 11405996 PMC8078195

[15] Heysieattalab S, Sadeghi L. Effects of delphinidin on pathophysiological signs of nucleus basalis of Meynert lesioned rats as animal model of Alzheimer disease. Neurochem Res. 2020;45(7):1636–1646. doi: 10.1007/s11064-020-03027-w 32297026

[16] Sawasdee P, Sabphon C, Sitthiwongwanit D, Kokpol U. Anticholinesterase activity of 7-methoxyflavones isolated from Kaempferia parviflora. Phytother Res. 2009;23(12):1792–1794. doi: 10.1002/ptr.2858 19548291

[17] Lopez CM, Govoni S, Battaini F, Bergamaschi S, Longoni A, Giaroni C, et al. Effect of a new cognition enhancer, alpha-glycerylphosphorylcholine, on scopolamine-induced amnesia and brain acetylcholine. Pharmacol Biochem Behav. 1991;39(4):835–840. doi: 10.1016/0091-3057(91)90040-9 1662399

[18] Lee HC, Fellenz-Maloney MP, Liscovitch M, Blusztajn JK. Phospholipase D-catalyzed hydrolysis of phosphatidylcholine provides the choline precursor for acetylcholine synthesis in a human neuronal cell line. Proc Natl Acad Sci USA. 1993;90(21):10086–10090. doi: 10.1073/pnas.90.21.10086 8234260 PMC47718

[19] Yu TX, Zhang P, Guan Y, Wang M, Zhen MQ. Protective effects of luteolin against cognitive impairment induced by infusion of Aβ peptide in rats. Int J Clin Exp Pathol. 2015;8(6):6740–6747. 26261557 PMC4525891

[20] Kimura J, Shimizu K, Takito J, Nemoto K, Degawa M, Yokosuka A, et al. Upregulatory effects of nobiletin, a citrus flavonoid with anti-dementia activity, on the gene expression of mAChR, ChAT, and CBP. Planta Med Lett. 2015;2(1):e12–14. doi: 10.1055/s-0035-1545937

[21] Paduszyńska A, Banach M, Rysz J, Dąbrowa M, Gąsiorek P, Bielecka-Dąbrowa A. Cytisine—from the past to the future. Curr Pharm Des. 2018;24(37):4413–4423. doi: 10.2174/1381612825666181123124733 30885113

[22] Seeger RC, Rayner SA, Banerjee A, Chung H, Laug WE, Neustein HB, et al. Morphology, growth, chromosomal pattern and fibrinolytic activity of two new human neuroblastoma cell lines. Cancer Res. 1977;37(5):1364–1371. 856461

[23] Richardson UI, Liscovitch M, Blusztajn JK. Acetylcholine synthesis and secretion by LA-N-2 human neuroblastoma cells. Brain Res. 1989;476(2):323–331. doi: 10.1016/0006-8993(89)91253-5 2702472

[24] Singh IN, Sorrentino G, McCartney DG, Massarelli R, Kanfer JN. Enzymatic activities during differentiation of the human neuroblastoma cells, LA-N-1 and LA-N-2. J Neurosci Res. 1990;25(4):476–485. doi: 10.1002/jnr.490250405 2352289

[25] Sandmann J, Wurtman RJ. Stimulation of phospholipase D activity in human neuroblastoma (LA-N-2) cells by activation of muscarinic acetylcholine receptors or by phorbol esters: relationship to phosphoinositide turnover. J Neurochem. 1991;56(4):1312–1319. doi: 10.1111/j.1471-4159.1991.tb11427.x 2002344

[26] Rylett RJ, Goddard S, Lambros A. Regulation of expression of cholinergic neuronal phenotypic markers in neuroblastoma LA-N-2. J Neurochem. 1993;61(4):1388–1397. doi: 10.1111/j.1471-4159.1993.tb13632.x 8376993

[27] Yamada T, Inazu M, Tajima H, Matsumiya T. Functional expression of choline transporter-like protein 1 (CTL1) in human neuroblastoma cells and its link to acetylcholine synthesis. Neurochem Int. 2011;58(3):354–65. doi: 10.1016/j.neuint.2010.12.011 21185344

[28] Perry M, Li Q, Kennedy RT. Review of recent advances in analytical techniques for the determination of neurotransmitters. Anal Chim Acta. 2009;653(1):1–22. doi: 10.1016/j.aca.2009.08.038 19800472 PMC2759352

[29] Murai S, Miyate H, Saito H, Nagahama H, Masuda Y, Itoh T. Simple determination of acetylcholine and choline within 4 min by HPLC-ECD and immobilized enzyme column in mice brain areas. J Pharmacol Methods. 1989;21(4):255–262. doi: 10.1016/0160-5402(89)90063-6 2547119

[30] Tucek S. Problems in the organization and control of acetylcholine synthesis in brain neurons. Prog Biophys Mol Biol. 1984;44(1):1–46. doi: 10.1016/0079-6107(84)90011-7 6385131

[31] Uutela P, Reinilä R, Piepponen P, Ketola RA, Kostiainen R. Analysis of acetylcholine and choline in microdialysis samples by liquid chromatography/tandem mass spectrometry. Rapid Commun Mass Spectrom. 2005;19(20):2950–2956. doi: 10.1002/rcm.2160 16180202

[32] Noori HR, Fliegel S, Brand I, Spanagel R. The impact of acetylcholinesterase inhibitors on the extracellular acetylcholine concentrations in the adult rat brain: a meta-analysis. Synapse. 2012;66(10):893–901. doi: 10.1002/syn.21581 22733599

[33] Lau JK, Brown KC, Dasgupta P. Measurement of acetylcholine from cell lines. Bio Protoc. 2013;3(24):e1007. doi: 10.21769/bioprotoc.1007 27390758 PMC4932865

[34] Welbat JU, Chaisawang P, Chaijaroonkhanarak W, Prachaney P, Pannangrong W, Sripanidkulchai B, et al. Kaempferia parviflora extract ameliorates the cognitive impairments and the reduction in cell proliferation induced by valproic acid treatment in rats. Ann Anat. 2016;206:7–13. doi: 10.1016/j.aanat.2016.04.029 27142346

[35] Food US and Administration Drug. Direct food substances affirmed as Generally Recognized as Safe (GRAS), Enzyme-Modified Lecithin, Code of Federal Regulations Title 21, Volume 3, 184.1063. 2020 Apr 1 [cited 2021 Sep 8]. Available from: https://www.accessdata.fda.gov/scripts/cdrh/cfdocs/cfcfr/CFRSearch.cfm?fr=184.1063

[36] Casamenti F, Scali C, Pepeu G. Phosphatidylserine reverses the age-dependent decrease in cortical acetylcholine release: a microdialysis study. Eur J Pharmacol. 1991;194(1):11–16. doi: 10.1016/0014-2999(91)90117-9 2060587

[37] Imran M, Rauf A, Abu-Izneid T, Nadeem M, Shariati MA, Khan IA, et al. Luteolin, a flavonoid, as an anticancer agent: A review. Biomed Pharmacother. 2019;112:108612. doi: 10.1016/j.biopha.2019.108612 30798142

[38] Nakajima A, Ohizumi Y, Yamada K. Anti-dementia activity of nobiletin, a citrus flavonoid: A review of animal studies. Clin Psychopharmacol Neurosci. 2014;12(2):75–82. doi: 10.9758/cpn.2014.12.2.75 25191498 PMC4153867

[39] Feuerstein TJ, Lehmann J, Sauermann W, van Velthoven V, Jackisch R. The autoinhibitory feedback control of acetylcholine release in human neocortex tissue. Brain Res. 1992;572(1–2):64–71. doi: 10.1016/0006-8993(92)90451-e 1611539

[40] Singh M, Kaur M, Kukreja H, Chugh R, Silakari O, Singh D. Acetylcholinesterase inhibitors as Alzheimer therapy: from nerve toxins to neuroprotection. Eur J Med Chem. 2013;70:165–188. doi: 10.1016/j.ejmech.2013.09.050 24148993

[41] Sperling RA, Donohue MC, Raman R, Sun CK, Yaari R, Holdridge K, et al. Association of factors with elevated amyloid burden in clinically normal older individuals. JAMA Neurol. 2020;77(6):735–745. doi: 10.1001/jamaneurol.2020.0387 32250387 PMC7136861

[42] Allgaier M, Allgaier C. An update on drug treatment options of Alzheimer’s disease. Front Biosci (Landmark Ed). 2014;19:1345–1354. doi: 10.2741/4285 24896354

[43] Leclerc M, Dudonné S, Calon F. Can natural products exert neuroprotection without crossing the blood-brain barrier? Int J Mol Sci. 2021;22(7):3356. doi: 10.3390/ijms22073356 33805947 PMC8037419

