# Supplementary material for: A novel in vitro assay model developed to measure both extracellular and intracellular acetylcholine levels for screening cholinergic agents
Source: PLoS One. 2021 Oct 12;16(10):e0258420. doi: 10.1371/journal.pone.0258420 (PMC8509891; doi:10.1371/journal.pone.0258420)
Supplement: S1 Table — (DOCX) [file pone.0258420.s001.docx]

**S1 Table. List of drug compounds and food constituents evaluated in our cell-based assay.**

| Name | Manufacturer | Concentration | Solvent |
| --- | --- | --- | --- |
| Arachidonic acid | Tokyo Chemical Industry Co., Ltd. | 20 µg/mL | Ethanol |
| Astaxanthin | Wako Pure Chemical Industries, Ltd. | 100 µM | Dimethyl sulfoxide |
| Black ginger extract | Maruzen Pharmaceuticals Co., Ltd. | 25–100 µg/mL | Dimethyl sulfoxide |
| L-Carnitine | Wako Pure Chemical Industries, Ltd. | 100 µM | Dimethyl sulfoxide |
| Choline chloride | Wako Pure Chemical Industries, Ltd. | 100 µM | Ethanol |
| Citrulline | Sigma-Aldrich | 100 µM | Distilled water |
| Curcumin | Wako Pure Chemical Industries, Ltd. | 20 µM | Dimethyl sulfoxide |
| Cyanidin chloride | ChromaDex, Inc. | 100 µM | Dimethyl sulfoxide |
| Cyanocobalamin | Wako Pure Chemical Industries, Ltd. | 100 µM | Distilled water |
| Cytisine | LKT Laboratories, Inc. | 50–5000 nM | Dimethyl sulfoxide |
| Delphinidin chloride | Tokiwa Phytochemical Co., Ltd. | 25–100 µM | Dimethyl sulfoxide |
| Docosahexaenoic acid | Cayman Chemical Company | 20 µg/mL | Ethanol |
| Eicosapentaenoic acid | Tokyo Chemical Industry Co., Ltd. | 20 µg/mL | Ethanol |
| Ferulic acid | Sigma-Aldrich | 100 µM | Dimethyl sulfoxide |
| Glycerophosphocholine | American Lecithin Company | 100 µg/mL | Distilled water |
| Glycine | Wako Pure Chemical Industries, Ltd. | 100 µM | Distilled water |
| Lutein | Wako Pure Chemical Industries, Ltd. | 100 µM | Dimethyl sulfoxide |
| Luteolin | Wako Pure Chemical Industries, Ltd. | 25–100 µM | Dimethyl sulfoxide |
| Lysophosphatidylcholine from egg yolk | Wako Pure Chemical Industries, Ltd. | 3.125–25 µg/mL | Ethanol |
| Methylcobalamin | Wako Pure Chemical Industries, Ltd. | 100 µM | Distilled water |
| (+)-Muscarine chloride | Sigma-Aldrich | 100 µM | Distilled water |
| Nobiletin | INDOFINE Chemical Company | 100 µM | Dimethyl sulfoxide |
| Octanoic acid | Wako Pure Chemical Industries, Ltd. | 100 µg/mL | Ethanol |
| Phosphatidylcholine from egg yolk | Wako Pure Chemical Industries, Ltd. | 100 µg/mL | Ethanol |
| Phosphatidylethanolamine, dimyristoyl | Wako Pure Chemical Industries, Ltd. | 100 µg/mL | Dimethyl sulfoxide |
| Phosphatidylethanolamine, dioleoyl | Wako Pure Chemical Industries, Ltd. | 100 µg/mL | Dimethyl sulfoxide |
| Phosphatidylethanolamine, dipalmitoyl | Wako Pure Chemical Industries, Ltd. | 100 µg/mL | Dimethyl sulfoxide |
| Phosphatidylethanolamine, distearoyl | Wako Pure Chemical Industries, Ltd. | 100 µg/mL | Dimethyl sulfoxide |
| Phosphatidylserine from soybean | Sigma-Aldrich | 100 µg/mL | Ethanol |
| Physostigmine | Sigma-Aldrich | 5 nM–50 µM | Dimethyl sulfoxide |
| Pyrroloquinoline quinone | Wako Pure Chemical Industries, Ltd. | 100 µM | Dimethyl sulfoxide |
| L-Serine | Wako Pure Chemical Industries, Ltd. | 100 µM | Distilled water |
| cis-15-Tetracosenoic acid | Tokyo Chemical Industry Co., Ltd. | 100 µg/mL | Ethanol |
| Zeaxanthin | EXTRASYNTHESE | 100 µM | Dimethyl sulfoxide |
