# Supplementary figures and images for: A novel in vitro assay model developed to measure both extracellular and intracellular acetylcholine levels for screening cholinergic agents
Source: PLoS One. 2021 Oct 12;16(10):e0258420. doi: 10.1371/journal.pone.0258420 (PMC8509891; doi:10.1371/journal.pone.0258420)

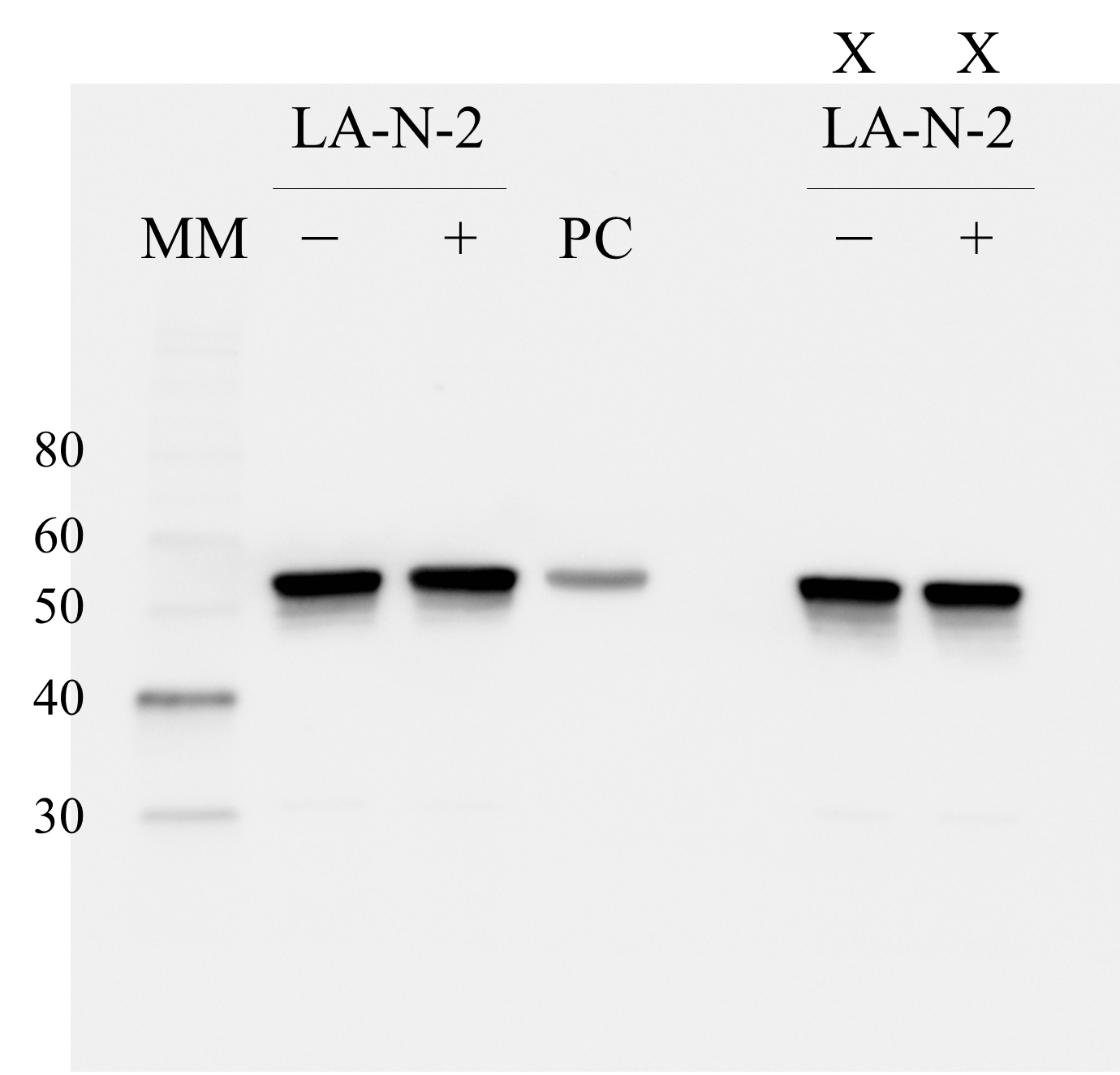

Supplement: S1 Raw image — Fig 1 was generated from this raw image. We evaluated different lysates of LA-N-2 cells that were harvested another day, and confirmed the reproducibility of the expression of mAChR M2. (TIF) [file pone.0258420.s003.tif]

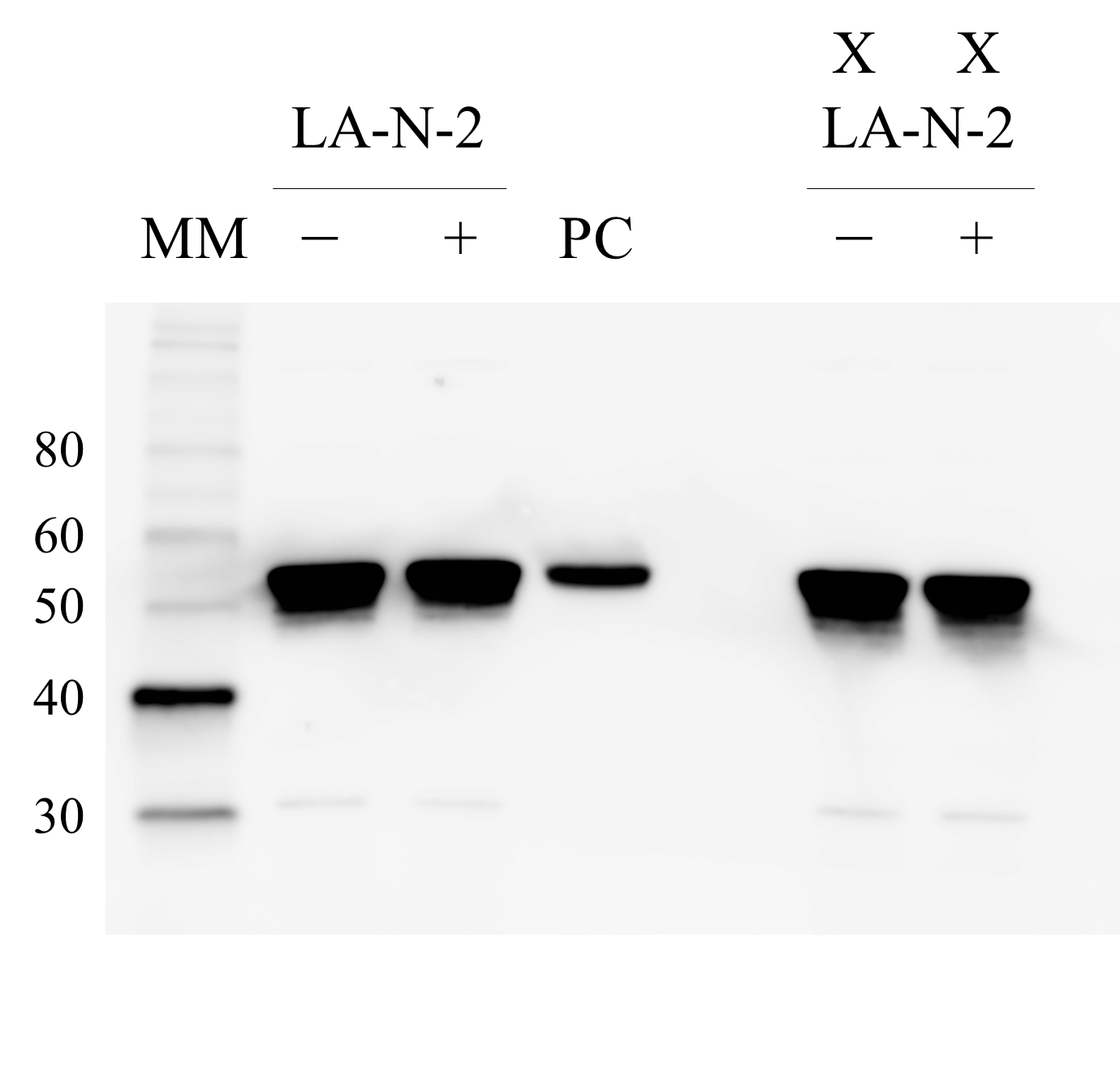

Supplement: S2 Raw image — The same membrane as S1 Raw image but with longer exposure time to increase the intensity of bands of the molecular weight marker. (TIF) [file pone.0258420.s004.tif]
