# Supplementary material for: A novel in vitro assay model developed to measure both extracellular and intracellular acetylcholine levels for screening cholinergic agents
Source: PLoS One. 2021 Oct 12;16(10):e0258420. doi: 10.1371/journal.pone.0258420 (PMC8509891; doi:10.1371/journal.pone.0258420)
Supplement: Attachment — Submitted filename: Response to Reviewers.doc [file pone.0258420.s005.doc]

**Our response to Journal Requirements**

The requirements and comments are shown in italics.

1) *Please ensure that your manuscript meets PLOS ONE's style requirements, including those for file naming.*

We apologize for the insufficient formatting. We have carefully read the Journal’s style templates again and revised the format.

2-i) *Please provide an amended Funding Statement declaring this commercial affiliation, as well as a statement regarding the Role of Funders in your study.*

The amended Funding Statement is: “The funder provided support in the form of salaries for authors [RT and KH], but did not have any additional role in the study design, data collection and analysis, decision to publish, or preparation of the manuscript. The specific roles of these authors are articulated in the ‘author contributions’ section.”

2-ii) *Please also provide an updated Competing Interests Statement declaring this commercial affiliation along with any other relevant declarations relating to employment, consultancy, patents, products in development, or marketed products, etc.*

The updated Competing Interests Statement is “I have read the journal's policy and the authors of this manuscript have the following competing interests: The authors are employees of Otsuka Pharmaceutical Co., Ltd. This does not alter our adherence to PLOS ONE policies on sharing data and materials.”

3) *Please amend either the title on the online submission form (via Edit Submission) or the title in the manuscript so that they are identical.*

We apologize for the inconsistency. The title on the online submission form has been amended and they are now identical.

4) *We note that you have included the phrase “data not shown” in your manuscript.*

5) *We note that you have indicated that data from this study are available upon request.*

We have included all additional data in the Supporting Information and excluded the phrase “data not shown” from the manuscript.

**Our response to Reviewers**

Reviewer #1

Thank you for your valuable comments and for suggesting some interesting research papers related to choline-containing phospholipids. We agree that the strategy targeting beta-amyloid and neurofibrillary tangles seems to be effective. However, we consider that adopting a multidirectional approach increases the possibility to successfully cure neurodegenerative disorders, a notion that is supported by a paper you suggested, which showed the enhanced effect of donepezil when combined with choline alphoscerate. The cholinergic agents found by our *in vitro* system could possibly be combined with other types of treatment agents, such as anti-amyloid-beta antibody. Further research is warranted to assess the effectiveness of such a combination.

Regarding the title of our manuscript, the approach we adopted has sufficient novelty in the space of phytomedicine and method development as no other screening assays that utilized this method have been reported. Therefore, we consider the original title appropriate.

Reviewer #2

1. *The author could draw a schematic diagram in one figure to illustrate (the principle of) this new assay. This would help readers to understand their manuscript.*

Thank you for your helpful advice. We drew and added a schematic diagram as Fig 1.

2. *Many food constituents have been tested in this assay under specific doses. Could authors discuss or mention whether relevant doses are similar to that of normal intake by human?*

We consider that concentrations adopted for some food constituents may be higher than those observed in the human blood and brain after their normal intake. However, we believe that the developed model can be used for preliminary screening because it could detect the change in ACh levels after treatment with some agents, the cholinergic activities of which have already been shown in *in vivo* or clinical studies.

Nevertheless, this limitation has been described in the Discussion (page 25, line 379-83).

3. *Based on the data you discussed, it seems that the roles of CHT1, ChAT, mAChR, and AChE cannot be distinguished when different drugs were applied (in your assay). Then, what degrees do you think the* *system you used could simulate the cholinergic metabolic system in physiological condition? Please discuss it.*

We believe that the discrete contributions of CHT1, ChAT, mAChR, and AChE on changes in ACh levels should be addressed in another study. The concentration of extracellular Ch in the assay was maintained between 3 and 4 µM over a 5-h incubation, which is consistent with the physiological concentration.

Relevant sentences have been added in the Discussion (page 21, line 271-7).

4. *I would suggest to replot at least some tables to graphs, in order for better visualization of your relevant data. There are simply too many tables.*

Given the different concentration ranges of ACh and Ch, we found that it was difficult to put these values in a single graph. In addition, we have added a concentration unit (µM) considering a comment by Reviewer #3, which made it more difficult to illustrate these results in graphs. Thus, we decided to leave them as tables. We hope you are satisfied with our opinion.

5. *In the discussion part, the authors stated, “Given that delphinidin and black ginger extract improve cognitive function in vivo [15, 28], an increase in the intracellular ACh levels could serve as an indicator of cognitive improvement.” It is simply too strong a statement in my opinion. The authors should weaken*

We have weakened the statement (page 22, line 308).

Reviewer #3

1. *LA-N-2 cells are not cholinergic by nature but they adapt some cholinergic properties (especially by up-regulation of ChAT) when differentiated e.g. by retinoic acid or LIF/CDF. It is understandable, therefore, that very little extracellular ACh is detected in most incubations because very little ACh is released by these cells, and the release machinery (SNAPs and SNAREs) may be defective. It would be interesting to see how the results change when the LA-N-2 cells were used in a differentiated state.*

*Cholinergic neurons are characterized by the expression of ChAT, CHT-1 and the vesicular ACh transporter (vAChT). Other cholinergic “features” listed by the authors are not exclusively cholinergic.*

We appreciate your detailed and constructive comments. As you pointed out, differentiation agents enhance cholinergic characteristics of LA-N-2. However, the cell line shows a partial cholinergic phenotype by nature and have been described as cholinergic elsewhere (ref. [24, 26]).

We also wondered if the results would change when LA-N-2 cells were differentiated. However, the cell line without differentiation seemed to produce a sufficient amount of ChAT for intracellular ACh to be produced and AChE for extracellular ACh to be degraded. Therefore, we prioritized a quick screen system set up and decided to perform assays without differentiation. This background has been added in the Discussion (page 21, line 279-84).

We apologize for the misleading description about “cholinergic” and corrected some sentences (page 2, line 11 and 22; page 4, line 40; and page 5, line 102 and 103).

2. *Unfortunately, data are not given as intra- and extracellular concentrations (µmol/L), and this should be corrected by the authors. Nevertheless, it appears that choline is concentrated much more highly in the medium, and this finding is at variance with physiological conditions: in the live brain, extracellular choline is approximately 3-4 µM whereas intracellular choline is approx. 50 µM. I wonder why choline is not adequately taken up by LA-N-2 cells: is there a lack of CHT-1 or of negative membrane potential? In addition to CHT-1, choline is also transported by low-affinity transporters from the family of organic cation transporters. I wonder if these are expressed in LA-N-2 cells? Also, where does the high choline level in the medium come from? Fetal calf serum maybe? Dead cells?*

We have recalculated Ch and ACh levels and expressed them as concentrations (µM). During this process, we found a mistake in our previous calculations, and have updated the values and reconfirmed statistical significance. Although critical aspects of the results did not change, we sincerely apologize for these necessary minor changes. Given the corrected values, we can observe that the concentrations of extracellular Ch are in good agreement with those in earlier studies (ref. [29, 30]). Regarding the concentration of intracellular Ch, the cellular contents were diluted as a result of lysing with 400 µL of perchloric acid as described in the Methods. Therefore, it is difficult to compare the values obtained in this study with a previously reported one (50 µM), and to judge whether Ch was not adequately taken up. Nevertheless, given that the concentration range of extracellular Ch obtained from the assays was similar to that in physiological conditions, we assumed that the transportation system for Ch worked in our model and did not conduct further research into it.

Although FBS contains Ch, the assay medium we used contained only 2% FBS. Therefore, we consider that the quantity of Ch derived from FBS is not significant. Dead cells were not observed under a microscope after the 5-h incubation without Ch.

3. *Cholinergic activations are due to an increase of extracellular ACh. Increases of intracellular ACh do not reflect cholinergic activation.*

We completely agree that the increase in intracellular ACh is not always parallel to cholinergic activation. Therefore, as a next step, an *in vivo* study should assess whether the treatment candidates chosen by this screening system affect cholinergic transmission.

4. *The next surprise is the poor efficacy of physostigmine in this model. Physostigmine only enhanced ACh levels at 5-50 µM concentration, and only by 10-15%. It must be noted that physostigmine is an AChE inhibitor with a short half-life of about 20 minutes in vivo, and most of its effect may have disappeared after 5 hours of incubation. A stronger response would have been observed with an inhibitor with a longer half-life on the enzyme, e.g. rivastigmine, or with an irreversibly acting organophosphate. I also wonder whether physostigmine (at high concentrations) interfered with the detection of ACh in the HPLC which is dependent on immobilized AChE in the enzyme reactor?*

Thank you again for your insightful comment. As a sentence has been added in the Discussion (page 21, line 288-90), 5-50 µM physostigmine is commonly used in microdialysis studies and we do not consider the efficacy of physostigmine observed in this study to be low. However, we agree that the amount of ACh detected in the extracellular fraction may increase if the incubation time is optimized for physostigmine or other AChE inhibitors with a longer half-life are applied. We consider that these possibilities should be assessed in another study.

We doubt the possibility that physostigmine interferes with AChE in the enzyme column because (a) the two columns preceding the enzyme column are packed with porous polymer, which would trap contaminants, including physostigmine in analytes, and (b) if so, we would observe an inverted concentration-response against ACh levels, which we did not.

5. *Nevertheless, it is noteworthy that only physostigmine, but none of the plant constituents was able to cause detectable levels of ACh in the medium. This indicates that physostigmine is able to inhibit the small amounts of AChE that may be secreted by LA-N-2 cells.*

*I do not know how the interaction of intracellular AChE with ACh would be in a neuroblastoma cell line.*

Although we did not investigate the interaction of intracellular AChE with ACh in LA-N-2 cells, the activity of intracellular AChE in another neuroblastoma cell line (N18TG2) was reported by Melone et al. (Int J Dev Neurosci. 1987;5(5-6):417-28), as well as the ability of the cell to release a considerable amount of the enzyme in the culture medium.

6. *The fact that choline at 100 µM increased intracellular choline ten-fold shows that high extracellular choline concentrations are required to increase intracellular choline – this speaks for a transport through a low-affinity carrier (see above). The fact that intracellular ACh also increased shows that intracellular choline was rate-limiting for ACh synthesis under basal conditions. It must be kept in mind that the test medium for the LA-N-2 cells was deficient in choline, a situation that does not occur in vivo – hence, addition of choline led to an immediate ACh synthesis. Before the addition of choline, the LA-N-2 cell could only use choline that was recovered from bound choline (PC mostly). To my knowledge, LA-N-2 cells are not able to synthesize choline de novo.*

It is indeed intriguing to further explore the Ch transport system in LA-N-2 cells. However, our focus was to develop a screening system to broadly catch cholinergic agents with several mechanisms of action. Hence, we generated a Ch-deficient environment on purpose to screen agents that can be a Ch source to the cell. We consider that it is worth screening Ch-containing compounds because positive clinical results have been reported for some of them.

7. *Such PLD-like activities are usually low in the body. Maybe they are present in LA-N-2 cells? Still, it is surprising that LA-N-2 cells do not express sufficient PLA2-activity to produce GPC and not enough phosphatase to break down GPC. It is less surprising that PC does not work because PC is rather stable, not water soluble and does not enter cells easily.*

PLD is present in synaptosomal membranes and it has been shown that LA-N-2 cells express PLD and PLA_2_ (ref. [24]). In the study, the authors found that the activities of GPC-phosphodiesterases in LA-N-2 cells do not change over incubation time, which suggests that the cell line is not able to use GPC as an ACh precursor.

Related sentences have been added in the Discussion (page 22, line 317-22).

8. *Muscarine increases ACh, this is surprising. Cytisin, a nicotinic agonist, does not. Which ACh receptors are expressed on LA-N-2 cells?*

Given your comment, we conducted western blotting and found muscarinic AChR M2 to be expressed on LA-N-2 cells.

Related sentences have been added in the Abstract, Methods, Results, and Discussion.

9. *The relevance of the data with plant constituents (e.g., flavonoids) is arguable. This reviewer does not believe that increases of ACh as observed in this study with luteolin or delphinidin would have a beneficial influence on a neurodegenerative disease such as Alzheimer´s dementia (AD). First, the extent of ACh increase is too low to affect cholinergic transmission to any measurable extent. Second, it is not known whether some of these constituents reach the blood or the brain in sufficient concentrations to exert an effect – flavonoids, for example, are extensively metabolized in the liver upon first pass. Experiments in cell culture do not reveal if substances are capable of crossing the blood-brain barrier. Taken together, it is very unlikely that delphinidin or luteolin would reach the high micromolar concentrations required to increase ACh in the brain in vivo.*

As you pointed out, luteolin, delphinidin, and other flavonoids are unlikely to reach the brain at a concentration of 100 µM after normal intake. However, we consider that it is still worth evaluating these food constituents even at relatively high concentrations as a preliminary screen. As stated in our manuscript, orally administered luteolin and delphinidin have been shown to improve the cholinergic system in the brain (ref. [15, 19]). Moreover, a technique to improve the bioavailability of unstable plant constituents has been recently developed. Using this technique, curcumin has been successfully used to improve AD pathology in a clinical trial (Ma Z, et al. J Control Release. 2019), and attempts to enhance the bioavailability of luteolin and other flavonoids are being made (Ali F, et al. CNS Neurol Disord Drug Targets. 2019).

We have added sentences to discuss the doses of food constituents we used in our study (page 25, line 379-83).
