# Supplementary material for: A novel in vitro assay model developed to measure both extracellular and intracellular acetylcholine levels for screening cholinergic agents
Source: PLoS One. 2021 Oct 12;16(10):e0258420. doi: 10.1371/journal.pone.0258420 (PMC8509891; doi:10.1371/journal.pone.0258420)
Supplement: Attachment — Submitted filename: Response to Reviewers.doc [file pone.0258420.s006.doc]

**Our response to Journal Requirements**

The requirements and comments are shown in italics.

*Please review your reference list to ensure that it is complete and correct. If you have cited papers that have been retracted, please include the rationale for doing so in the manuscript text, or remove these references and replace them with relevant current references. Any changes to the reference list should be mentioned in the rebuttal letter that accompanies your revised manuscript.*

We do not cite any retracted article in our manuscript. During the peer review and revision process, we have changed or removed some parts of the Introduction and Discussion. Along with these changes, we have removed some cited papers from the revised manuscript as they did not seem necessary to support our conclusions (Giacobini E, 2003; Nilsson Å, et al, 2019; Volpicelli LA, et al, 2004; Abreu-Villaça Y, 2011). Additionally, we have added some papers in response to the reviewers’ suggestions to improve the quality of our manuscript (their current reference numbers are 14, 26, 27, 30, 31, 32, 33, 39, and 43).

**Our response to Reviewers**

Reviewer #2

1. *The author applied many chemicals to the cell system using a series of concentrations. Will some high concentrations of the chemicals have toxicities towards cells and affect their normal metabolism? It would be better if you can discuss it in your manuscript.*

Thank you for your helpful advice. When the cells were treated with some chemicals including LPC at high concentrations, cell death and a decrease in ACh levels were observed. The related sentence and data have been added (page 22, line 318, and S1 dataset).

2. *Discussing the expression of receptor subtypes on the membrane based on relevant RNA-seq data will be helpful.*

We searched for the RNA-seq and array data regarding the expression of ACh reseptors in LA-N-2 cells but could not find any (only short RNA-seq data is available). We consider that the expression of the other subtypes of mAChR should be investigated in another study.

Reviewer #3

1. *Line 40, the point that “the cholinergic system contains choline” is trivial. Choline is present all over the body, in blood plasma and in all cells. Please delete. It would be more reasonable to include acetylcholine as a characteristic of the cholinergic system (which was named cholinergic system out of reluctance to write “acetylcholinergic system”).*
2. *Line 46. Similarly, there is no evidence that lack of choline plays a role in cholinergic dysfunction or dementia, except in experimental systems. Please write “agonistic effects at/on receptors”, not “against”.*

Although many clinical trials that administered Ch sources failed to improve the clinical status of patients with dementia, some Ch sources combined with an AChE inhibitor have succeeded and showed better outcomes than the AChE inhibitor alone (Traini E, et al. 2020, Piamonte BLC, et al. 2020). Therefore, we consider that detecting the Ch providing property of test compounds is of value and that our screening system enables it, at least for some Ch containing compounds. We consider this as one of the advantages of our system. Therefore, we did not delete the sentences you pointed out, but modified them (page 2, line 11 and page 3, line 40). In addition, we have changed the preposition from “against” to “at” (page 3, line 47).

3. *Line 289: The Lau et al. study does not seem to make much sense. An increase of ACh with neostigmine at 50 µM, but not at 20 µM? Something is wrong there, in almost all labs neostigmine is active even at 1 µM.*

We believe that you may be thinking of the microdialysis study with neostigmine like reviewed in [32]. In the setting, 1 µM neostigmine is enough to detect ACh because ACh is protected from AChE once it goes through the semi-permeable membrane of the microdialysis probe. However, in Lau’s and our *in vitro* system, ACh is subjected to hydrolysis by AChE unless the enzyme is fully inhibited or deactivated (we added perchloric acid to the medium).

4. *Line 310: the fact that there is an increase of intracellular choline after addition of choline does not mean that there is CHT-1. Any choline transporter could do that. The authors may check the presence of CHT-1 by Western blot, or they can use hemicholinium-3 to get more information.*

We admit that we could not clearly distinguish CHT-1 from other types of Ch transporters. According to a previous study [27], the choline-uptake mechanism of LA-N-2 involves intermediate-affinity transporters (Ch transporter-like protein 1) rather than high-affinity ones (CHT-1) (they used hemicholinium-3 in their experiments). This finding explains why a relatively low incorporation of extracellular Ch was observed in our experimental setting. The related sentence has been added to the Discussion (page 22, line 316).

5. *Line 316: Since there is GPC diesterase, GPC should be useable by the cells; however, the enzyme is intracellular, and GPC is not taken up by cells. A change of GPC diesterase activity is not necessary to break down GPC, its presence is enough. Please rephrase the discussion of Singh et al. so that it makes sense. Lyso-PC is a membrane detergent and may have entered the cell by unspecific mechamisms, requiring no transporters.*

We have revised the sentence (page 23, line 321). Additionally, we agree that LPC is a membrane detergent and that this property may have contributed to the higher ACh accumulation after treatment with LPC than after treatment with Ch. Related sentences have been added to the Discussion (page 22, line 317).

6. *Line 317: The esterase does not “hydrate” GPC, it “hydrolyzes” GPC. Please change.*

We have corrected the sentence (page 22, line 323). We appreciate all of your excellent comments and suggestions.
